# Distinct Activities of Gli1 and Gli2 in the Absence of Ift88 and the Primary Cilia

**DOI:** 10.3390/jdb7010005

**Published:** 2019-02-19

**Authors:** Yuan Wang, Huiqing Zeng, Aimin Liu

**Affiliations:** 1 Department of Biology, Eberly College of Sciences, Center for Cellular Dynamics, Huck Institute of Life Science, The Penn State University, University Park, PA 16802, USA; yxw478@psu.edu (Y.W.); huz4@psu.edu (H.Z.); 2 Department of Occupational Health, School of Public Health, China Medical University, No.77 Puhe Road, Shenyang North New Area, Shenyang 110122, China

**Keywords:** Hh signaling, Shh, neural tube, patterning, intraflagellar transport, Gli3, Sufu, Smo, mouse

## Abstract

The primary cilia play essential roles in Hh-dependent Gli2 activation and Gli3 proteolytic processing in mammals. However, the roles of the cilia in Gli1 activation remain unresolved due to the loss of *Gli1* transcription in cilia mutant embryos, and the inability to address this question by overexpression in cultured cells. Here, we address the roles of the cilia in Gli1 activation by expressing *Gli1* from the *Gli2* locus in mouse embryos. We find that the maximal activation of Gli1 depends on the cilia, but partial activation of Gli1 by Smo-mediated Hh signaling exists in the absence of the cilia. Combined with reduced Gli3 repressors, this partial activation of Gli1 leads to dorsal expansion of V3 interneuron and motor neuron domains in the absence of the cilia. Moreover, expressing *Gli1* from the *Gli2* locus in the presence of reduced Sufu has no recognizable impact on neural tube patterning, suggesting an imbalance between the dosages of Gli and Sufu does not explain the extra Gli1 activity. Finally, a non-ciliary Gli2 variant present at a higher level than Gli1 when expressed from the *Gli2* locus fails to activate Hh pathway ectopically in the absence of the cilia, suggesting that increased protein level is unlikely the major factor underlying the ectopic activation of Hh signaling by Gli1 in the absence of the cilia.

## 1. Introduction

The Hedgehog (Hh) family of signaling molecules underlies numerous developmental processes and malignancies in humans and mice [1]. Hh signaling in mammals requires the primary cilia, a cell surface organelle present in almost all post-mitotic cells in the mammalian body [2]. The glioma-associated oncogene homolog (Gli) family of transcription factors mediates the transcriptional response of Hh signaling, and all three members of the family are localized to the tips of the cilia upon Hh stimulation [3,4]. Given the importance of Hh signaling and the primary cilia in development and diseases, it is critical to understand the roles of the cilia in the activation of Gli proteins.

Sonic hedgehog (Shh), one of the Hh family members, is secreted from cells of the notochord and the floor plate, a group of glia at the ventral midline of the neural tube, and induces various cell fates in the ventral neural tube [1]. Loss of *Shh* results in the complete loss of ventral cell types including the floor plate, V1, V2 and V3 interneurons and motor neurons [5]. Gli2 is the primary activator downstream of Shh and is essential for the fates of the floor plate and V3 interneurons [6,7]. Gli3 plays a predominantly negative role in Hh signaling, and removing Gli3 restores motor neurons in *Shh;Gli3* double mutant neural tube [8]. *Gli1* expression is dependent on Gli2 and Gli3, and loss of *Gli1* does not disrupt mouse development [9,10,11]. However, loss of *Gli1* leads to defects in Shh pathway activation and ventral neural tube development when one copy of *Gli2* is removed, suggesting that it contributes to a threshold of Gli activator activity required for the full activation of the Shh pathway [9]. More importantly, Gli1 appears to be critical in pathogenesis of multiple types of malignancies, hence understanding the mechanism of its activation is clinically important [12,13,14,15].

The requirement for the cilia in Hh signaling was first revealed by the loss of floor plate and V3 interneurons, as well as reduced Hh target gene expression, in a few mutants that fail to grow cilia [16]. Specifically, both the activation of full-length Gli2 and the generation of Gli3 repressor through proteolytic processing are dependent on the cilia (e.g., [17,18,19]). We recently showed that removing Gli2 from the tips of the cilia prevents its Hh-dependent activation, further confirming the critical role of cilia in Gli2 activation [20].

Suppressor of fused (Sufu) is an essential negative regulator of Hh signaling in mammals, loss of which results in severe disruption of embryonic development including extreme ventralization of the neural tube [21,22]. Our previous double and triple mutant analyses indicated that all three Gli proteins underlie the extreme Hh pathway activation in *Sufu* mutants [23]. Biochemical analyses suggested that Sufu acts through direct physical interaction with Gli proteins, both in the cytoplasm and inside the nucleus [24,25,26,27]. Interestingly, loss of *Sufu* in the absence of the cilia leads to the over activation of Hh pathway, suggesting that the roles of the cilia in Hh signaling is to mediate the Hh-induced alleviation of repression on Gli proteins by Sufu [28,29]. Subsequent biochemical studies showed that separation between Sufu and Gli proteins was indeed dependent on the cilia [30,31].

Although the roles of the primary cilia in Gli2 activation and Gli3 processing have been elucidated, whether the activation of Gli1 is dependent on the cilia remains enigmatic. *Gli1* transcription is severely reduced in cilia mutants, precluding the direct analysis of the roles of the cilia in Gli1 activation with these mutants [16,18]. The roles of cilia in Gli1 activation cannot be revealed by overexpressing *Gli1* in cultured cilia mutant cells either, as insufficient Sufu is present in the cells to antagonize the activity of overexpressed Gli1, making it constitutively active independent of Hh signaling input and the primary cilia [28,29]. In the current study, we test the roles of the cilia in Gli1 activation by expressing *Gli1* at a physiological level from the *Gli2* locus (*Gli2^1ki^*) in cilia mutants. We find that loss of cilia prevented the maximal activation of Gli1 and the formation of the floor plate. Surprisingly, Gli1 was partially activated in the absence of the cilia, resulting in drastic dorsal expansion of the V3 interneuron and motor neuron domains. We show that expressing *Gli1* from the *Gli2* locus leads to increased motor neuron formation with reduced Gli3 dosage, suggesting that compromised Gli3 repressor production in the absence of cilia may contribute to the partial activation of Hh signaling in the neural tube when *Gli1* is expressed from the *Gli2* locus in the absence of the cilia. This cilia-independent activation of Gli1 is dependent on Hh signaling because expressing *Gli1* from the *Gli2* locus does not change neural tube patterning in the absence of *Smo*. Furthermore, *Gli1* expression from the *Gli2* locus did not alter neural tube patterning with reduced dosage of *Sufu*, suggesting it did not activate Hh pathway by changing the balance between dosages of Gli and Sufu proteins. Finally, replacing endogenous *Gli2* with a stable, non-ciliary form of *Gli2*, *Gli2^∆CLR^*, failed to induce ectopic V3 interneurons and motor neurons in the absence of the cilia, suggesting that the cilia-independent Gli1 activity was not simply the result of higher protein level. In summary, we show different degrees of dependence of Gli1 and Gli2 on the cilia for their activation, suggesting that blocking ciliogenesis may not inhibit malignancies caused by aberrant Gli1 activation.

## 2. Materials and Methods

### 2.1. Animals

Mutant mouse strains used in this study include *Gli2^tm3(Gli1)Alj^* (a.k.a *Gli2^1kie^*) [32], *Gli2^tm3.1(Gli1)Alj^* (a.k.a. *Gli2^1ki^*) [32], Gli2^tm1.1(Gli2*)Aliu^ (a.k.a. *Gli2^∆CLRki^*) [20], *Ift88^tm1Rpw^* [33], *Gli3^Xt-J^* [34], *N-Tg(EIIa-cre)C5379Lmgd/J* [35], and *Sufu^tm1Rto^* [22], and were genotyped as previously published. All animals were kept on 129/SvPasCrl (Charles River Laboratories) background. The use of the animals in this work was approved by the Institutional Animal Care and Use Committee at the Penn State University.

### 2.2. Immunofluorescence Assay on Cryosections

Mouse embryos were fixed in 4% paraformaldehyde (PFA) in phosphate-balanced saline (PBS) for up to 1 h, washed with PBS briefly and left in 30% sucrose overnight, embedded in O.C.T freezing media and frozen at −80 °C. Cryosections at 10 µm thickness were cut with a Leica LM1900 Cryostat. For immunofluorescence assays, sections were allowed to dry at room temperature for 1 h, blocked in blocking buffer (PBS plus 0.1% Triton X-100 and 1% goat serum). They were then incubated in blocking buffer with appropriate primary antibodies at 4 °C overnight, washed in blocking buffer three times and incubated in blocking buffer with Cy3-conjugated secondary antibodies, wash three more times and mounted with DABCO (Sigma-Aldrich, Saint Louis, MO, USA). Antibodies used: Foxa2, Nk2.2, Nkx6.1, Pax6, Pax7, Shh (DSHB) and Olig2 (Millipore, AB9610). Photos were taken on a Nikon E600 microscope with a Micropublisher CCD camera (QImaging, Surrey, BC, Canada).

### 2.3. Immunoblot Analyses

Whole-cell protein lysates were prepared, separated on SDS polyacrylamide gel and transferred to nitrocellulose membrane according to a previously described protocol [36]. After primary antibody incubation, membranes were incubated with IRD680- and IRD800-conjugated secondary antibodies (LI-COR), and scanned on a LICOR Odyssey CLx imaging system. Antibodies against FLAG and β-tubulin were purchased from Sigma-Aldrich. Quantitative analyses were performed using NIH Image J.

### 2.4. RNA In Situ Hybridization on Cryosections

Embryos were fixed in 4% PFA at 4 °C overnight, washed in DEPC-treated PBS and processed for cryosection. RNA in situ hybridization with Digoxigenin-labeled riboprobes against *Gli1* was performed on the transverse sections through the neural tube according to the protocol described in [37]. Photos were taken on a Nikon E600 microscope with a Micropublisher CCD camera.

## 3. Results

### 3.1. Gli1 Expression Was Uncoupled from Hh Signaling in Gli2^1ki^ Embryos

More than a decade of research has started to reveal the essential roles of the primary cilia in the activation of Gli2 [16,20,28,29] and proteolytic processing of Gli3 [17,18,19] in mammals. However, how the Gli1 protein responds to the loss of cilia remains an open question because *Gli1* expression is dependent on Hh signaling and is greatly reduced in the absence of the cilia [18]. We and others also showed that an overexpression approach was not appropriate for addressing this question as it rendered both Gli1 and Gli2 cilia-independent due to the disruption of the stoichiometry between Gli proteins and their direct inhibitor Sufu [28,29]. Therefore, the only proper way to address this question is to express *Gli1* at a physiological level independent of Hh pathway activation.

**Figure 1:**
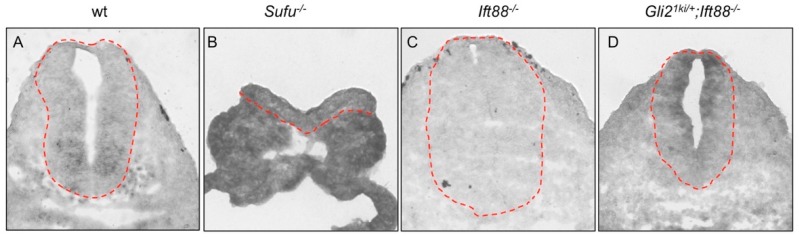
Expression of *Gli1* from the *Gli2* locus was independent of the primary cilia. RNA in situ hybridization images of transverse sections through the E10.5 neural tubes. (**A**) *Gli1* was expressed in a ventral-to-dorsal gradient in the wild type (wt) neural tube. (**B**) Widespread *Gli1* expression was found in *Sufu*^−/−^ mutant embryos. (**C**) *Gli1* expression was greatly reduced in *Ift88*^−/−^ mutant embryos. (**D**) *Gli1* expression was present in all progenitor cells in the *Gli2^1ki^*^/+^*;Ift88*^−/−^ double mutant neural tubes. Dashed lines outline the neural tubes. *n* = 3 embryos for each genotype. Gli: Glioma-associated oncogene homolog.

To achieve such a goal, we took advantage of a *Gli2^1ki^* mouse strain in which the *Gli1* open reading frame was inserted into the first coding exon of *Gli2* [32]. Bai and Joyner (2001) showed that *Gli1* was expressed in the same pattern as *Gli2* in this knock-in animal [32]. To determine whether *Gli1* expression became cilia-independent in *Gli2^1ki^* embryos, we performed RNA in situ hybridization analyses. As reported, *Gli1* was expressed in a ventral-to-dorsal gradient in the wild type neural tube (Figure 1A) [38,39]. As a control, *Sufu* mutant embryos exhibited widespread high levels of *Gli1* expression (Figure 1B) [22]. Consistent with compromised Hh signaling activity in the absence of the cilia, *Gli1* expression was greatly reduced in the *Ift88* mutant neural tube that failed to grow cilia (Figure 1C) [16,33]. In contrast, *Gli1* was expressed throughout the progenitors of the neural tube in *Gli2^1ki^*^/+^*;Ift88*^−/−^ double mutants, suggesting that *Gli1* expression from the *Gli2* locus was independent of the cilia (Figure 1D). These data indicated that *Gli1* expression from the *Gli2* locus was independent of Hh signaling and the presence of cilia, making it possible to analyze the roles of cilia in Gli1 activation.

### 3.2. The Cilia Are Essential for Maximal Activation of Gli1

**Figure 2:**
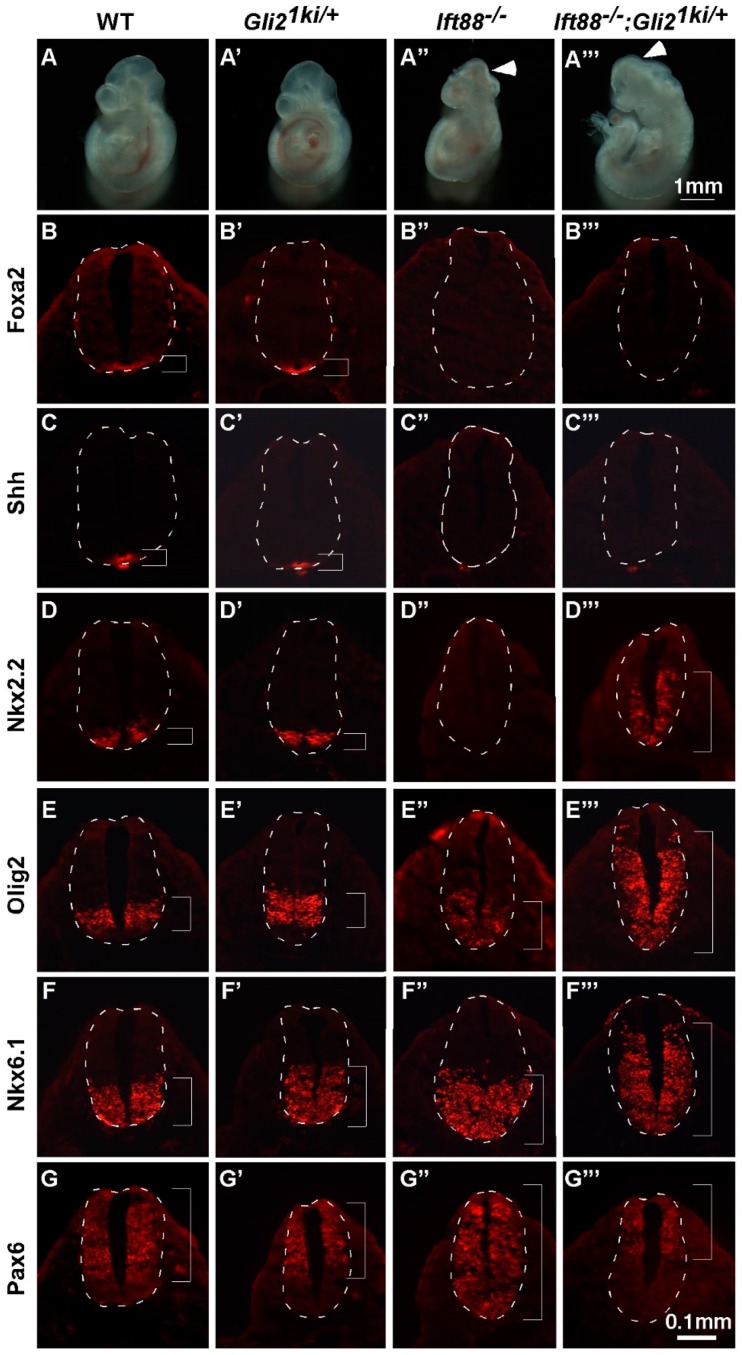
Partial activation of Gli1 in the absence of cilia. (**A**–**A”’**) Lateral views of E10.5 embryos. Wild type (**A**) and *Gli2^1ki^*^/+^ (**A’**) embryos look similar. *Ift88*^−/−^ (**A”**) and *Gli2^1ki^*^/+^;*Ift88*^−/−^ (**A”’**) embryos exhibit exencephaly (arrowheads) and twisted body axes. (**B**–**G”’**) Transverse sections of E10.5 embryos processed for immunofluorescence analyses. (**B**–**B”’**) *Foxa2* was expressed in the floor plates of the wild type (**B**) and *Gli2^1ki^*^/+^ (**B’**) neural tubes. It was absent in the *Ift88^−^* (**B”**) and *Gli2^1ki^*^/+^;*Ift88*^−/−^ (**B”’**) neural tubes. (**C**–**C”’**) Shh protein was present in the notochords and ventral neural tubes of wild type (**C**) and *Gli2^1ki^*^/+^ (**C’**) embryos. In *Ift88*^−/−^ (**C”**) and *Gli2^1ki^*^/+^;*Ift88*^−/−^ (**C”’**) embryos, Shh was present in the notochords, but not in the neural tubes. (D-D”’) *Nkx2.2* labels V3 interneurons in the wild type (**D**) and *Gli2^1ki^*^/+^ (**D’**) neural tubes. It was absent in the *Ift88*^−/−^ (**D”**) neural tube. The *Nkx2.2* expression domain was expanded both ventrally and dorsally in the *Gli2^1ki^*^/+^;*Ift88*^−/−^ (**D”’**) neural tube. (**E**–E**”’**) *Olig2* labels motor neuron progenitors in the wild type (**E**) and *Gli2^1ki^*^/+^ (**E’**) neural tubes. It was expanded ventrally in the *Ift88*^−/−^ (**E”**) neural tube. The *Olig2* expression domain was expanded both ventrally and dorsally in the *Gli2^1ki^*^/+^;*Ift88*^−/−^ (**E”’**) neural tube. (**F**–**F”’**) *Nkx6.1* labels progenitors of V1-3 interneurons, motor neurons and the floor plate in the wild type (**F**) and *Gli2^1ki^*^/+^ (**F’**) neural tubes. It appears normal in the *Ift88*^−/−^ (**F”**) neural tube. The *Nkx6.1* expression domain was expanded dorsally in the *Gli2^1ki^*^/+^;*Ift88*^−/−^ (**F”’**) neural tube. (**G**–**G”’**) *Pax6* was expressed in the dorsal and lateral regions of the wild type (**G**) and *Gli2^1ki^*^/+^ (**G’**) neural tubes. It was expanded ventrally in the *Ift88*^−/−^ (**G”**) neural tube. *Pax6* expression was restricted more dorsally in the *Gli2^1ki^*^/+^;*Ift88*^−/−^ (**G”’**) neural tube. Dashed lines outline the neural tubes and brackets mark the expression domains. *n* = 3 embryos for each genotype.

Bai and Joyner (2001) showed that expressing *Gli1* from the *Gli2* locus rescued most aspects of embryonic development in the absence of *Gli2*, suggesting functional redundancy between these two proteins [32]. We hypothesized that if Gli1 activation was independent of the cilia, expressing *Gli1* from the *Gli2* locus should rescue embryonic development in the absence of the cilia. To test this hypothesis, we crossed *Gli2^1ki^* mice to *Ift88* mutants. At E10.5, *Gli2^1ki^*^/+^ embryos looked normal, consistent with a previous report (Figure 2A,A’; [32]). *Ift88* null mutants exhibited frequent exencephaly and twisted body (Figure 2A”). *Gli2^1ki^*^/+^*;Ift88*^−/−^ double mutants also exhibited frequent exencephaly and twisted body (Figure 2A”’). The failure to rescue the *Ift88* mutant phenotype appears to suggest that Gli1 activation is under the influence of the primary cilia.

To better determine the roles of cilia in Gli1 activation, we examined the dorsal/ventral (D/V) patterning of the neural tube at both the thoracic (anterior) and lumbar (posterior) levels. As similar neural tube patterning changes were present at both levels, we will focus on the results at the thoracic level. The floor plate at the ventral midline of the wild type neural tube expresses *Foxa2* at E10.5 (Figure 2B). Confirming a previous report [32], we found that *Foxa2* was expressed properly in the *Gli2^1ki^*^/+^ neural tube (Figure 2B’). *Foxa2* expression was absent in the *Ift88* mutant neural tube due to compromised Gli2 activation in the absence of the cilia (Figure 2B”; [16]). *Foxa2* expression was also absent in *Gli2^1ki^*^/+^*;Ift88*^−/−^ double mutants, suggesting that the maximal activation of Gli1 was dependent on the cilia (Figure 2B”’). Shh was produced in the notochord and floor plate in the wild type and *Gli2^1ki^*^/+^ neural tubes (Figure 2C,C’). Consistent with the loss of floor plates, Shh was present in the notochords, but not in the ventral neural tubes in the *Ift88* mutant (Figure 2C”) and *Gli2^1ki^*^/+^*;Ift88*^−/−^ double mutant (Figure 2C”’) neural tubes.

### 3.3. Gli1 Was Partially Activated in the Absence of Cilia

*Nkx2.2*-expressing V3 interneurons and their progenitors were adjacent to the floor plate and required lower levels of Shh signaling than those required for floor plate (Figure 2D). The number and location of V3 interneurons were not altered in *Gli2^1ki^*^/+^ mutants as previously reported (Figure 2D’) [32]. Consistent with an essential role of cilia in Gli2 activation, these neurons were completely absent in *Ift88* null mutants (Figure 2D”). To our surprise, the domain of V3 interneurons was not only present, but also greatly expanded dorsally in the *Gli2^1ki^*^/+^*;Ift88*^−/−^ double mutant neural tube, implying an increase in Hh pathway activity (Figure 2D”’). Motor neuron progenitors expressing *Olig2* were dorsal to V3 interneuron progenitors in both the wild type and *Gli2^1ki^*^/+^ neural tubes, and required moderate activation of the Hh pathway (Figure 2E,E’). *Olig2* expression was expanded to the ventral midline in the *Ift88*^−/−^ mutant neural tube, consistent with the loss of floor plate and V3 interneurons (Figure 2E”). The motor neuron domain was expanded both ventrally and dorsally in *Gli2^1ki^*^/+^*;Ift88*^−/−^ neural tube, suggesting that although maximal activation of Hh signaling was not achieved, an intermediate level of Hh pathway activation was present in more cells than in the wild type neural tube (Figure 2E”’). *Nkx6.1* labeled ventral half of the neural tube spanning the floor plate to V1 interneuron progenitors in the wild type, *Gli2^1ki^*^/+^ and *Ift88*^−/−^ single mutant neural tubes (Figure 2F–F”). Consistent with more widespread intermediate Hh pathway activation, the domain of *Nkx6.1* expression was expanded dorsally in the *Gli2^1ki^*^/+^*;Ift88*^−/−^ double mutant neural tube (Figure 2F”’). Finally, *Pax6* was expressed in the dorsal and lateral parts of the wild type and *Gli2^1ki^*^/+^ neural tubes (Figure 2G,G’), and was expanded ventrally in *Ift88*^−/−^ neural tube as a result of compromised Hh pathway activation (Figure 2G”). *Pax6* expression was limited to the dorsal half of the *Gli2^1ki^*^/+^*;Ift88*^−/−^ double mutant neural tube, reflecting increased Hh pathway activation in the ventral and lateral regions of the neural tube (Figure 2G”’). In summary, we found that Gli1 could be partially activated in the absence of the cilia.

### 3.4. Ectopic Gli1 Partially Ventralized the Neural Tube in the Presence of Reduced Gli3

**Figure 3:**
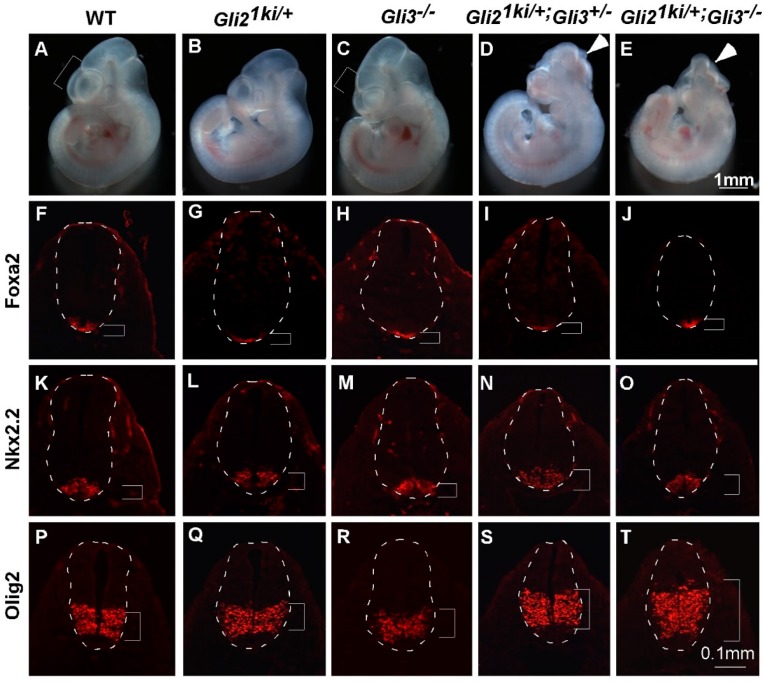
Gli3 antagonized Gli1 activity in the Gli21ki neural tube. (**A**–**E**) Lateral views of E10.5 embryos. Wild type (**A**) and *Gli2^1ki^*^/+^ (**B**) embryos looked similar. *Gli3*^−/−^ embryo (**C**) exhibited reduced telencephalon (bracket, compare to the one in **A**). *Gli2^1ki^*^/+^;*Gli3*^+/−^ (**D**) and *Gli2^1ki^*^/+^;*Gli3*^−/−^ (**E**) embryos exhibit exencephaly (arrowheads). (**F**–**T**) Transverse sections of E10.5 embryos processed for immunofluorescence analyses. Dashed lines outline the neural tubes and brackets mark the expression domains. (**F**–**J**) *Foxa2* was expressed in the floor plates of the wild type (**F**), *Gli2^1ki^*^/+^ (**G**), *Gli3*^−/−^ (**H**), *Gli2^1ki^*^/+^;*Gli3*^+/−^ (**I**) and *Gli2^1ki^*^/+^;*Gli3*^−/−^ (**J**) neural tubes. (**K**–**O**) *Nkx2.2* labels V3 interneurons in the wild type (**K**), *Gli2^1ki^*^/+^ (**L**), *Gli3*^−/−^ (**M**), *Gli2^1ki^*^/+^;*Gli3*^+/−^ (**N**) and *Gli2^1ki^*^/+^;*Gli3*^−/−^ (**O**) neural tubes. (**P**–**T**) *Olig2* labels motor neuron progenitors in the wild type (**P**), *Gli2^1ki^*^/+^ (**Q**) and *Gli3*^−/−^ (**R**) neural tubes. The *Olig2* expression domain was expanded dorsally in the *Gli2^1ki^*^/+^;*Gli3*^+/−^ (**S**) neural tube, and the expansion was more drastic in the *Gli2^1ki^*^/+^;*Gli3*^−/−^ (**T**) neural tube. *n* = 3 embryos for each genotype unless otherwise mentioned in the text.

Paradoxically, the neural tube patterning indicated that more cells in the *Gli2^1ki^*^/+^*;Ift88*^−/−^ neural tube experienced intermediate levels of Hh pathway activation than those of the *Gli2^1ki^*^/+^ neural tube, suggesting a negative role of the cilia in Hh pathway activation. As it was known that the cilia were essential for the proteolytic processing of Gli3 [18,19,30], we hypothesized that the reduction in Gli3 repressor activity contributed to the increase in Hh pathway activation in lateral regions of the *Gli2^1ki^*^/+^*;Ift88*^−/−^ neural tube. To test this hypothesis, we crossed *Gli2^1ki^*^/+^ mice to *Gli3*^+/−^ mice to generate *Gli2^1ki^*^/+^*;Gli3*^+/−^ double mutant embryos. At E10.5, *Gli2^1ki^*^/+^ and *Gli3*^+/−^ embryos were indistinguishable from wild type embryos (Figure 3A,B and data not shown). *Gli3*^−/−^ embryos exhibited smaller telencephalon (compare the brackets in Figure 3A,C; *n* = 4/6) and occasional exencephaly (data not shown; *n* = 2/6). Interestingly, *Gli2^1ki^*^/+^*;Gli3*^+/−^ double mutants exhibited frequent exencephaly (Figure 3D; *n* = 4/7), suggesting strong genetic interaction between ectopic *Gli1* expression and reduced Gli3 dosage in the patterning and/or proliferation of the brain. We further analyzed the neural tube patterning and found that the size and location of the floor plate and V3 interneuron domains in the *Gli2^1ki^*^/+^*;Gli3*^+/−^ double mutant neural tube were similar to those in the wild type, *Gli2^1ki^*^/+^ and *Gli3*^−/−^ neural tubes (Figure 3F–I,K–N). However, we observed a moderate dorsal expansion of the motor neuron progenitor domain in the *Gli2^1ki^*^/+^*;Gli3*^+/−^ double mutant neural tube (Figure 3S, compared to Figure 3P–R), suggesting that reducing Gli3 repressor activity did allow ectopically expressed Gli1 to moderately ventralize the lateral part of the neural tube through activating lower levels of Hh signaling.

*Gli2^1ki^*^/+^*;Gli3*^+/−^ pups were sickly and died shortly after weaning, preventing further breeding. *Gli2^1kie^* was similar to *Gli2^1ki^*, but contained a floxed neo cassette interfering with the expression of *Gli1* from the *Gli2* locus [32]. *Gli2^1kie^*^/+^*;Gli3*^+/−^ male were viable and fertile. Therefore, we crossed these mice with *Gli3*^+/−^*;EIIaCre* female mice to obtain *Gli2^1kie^*^/+^*;Gli3*^−/−^*;EIIaCre* embryos. As *EIIaCre* was expressed ubiquitously in the embryos [35], removing the floxed neo cassette and restoring the full expression of *Gli1* from the *Gli2* locus, these embryos were equivalent to *Gli2^1ki^*^/+^*;Gli3*^−/−^. We found that these embryos also exhibited frequent exencephaly (Figure 3E; *n* = 5/5). Although the floor plate and V3 interneurons were defined properly in these mutants (Figure 3J,O), the motor neuron progenitor domain was drastically expanded dorsally in the neural tube of these embryos (Figure 3T). These data suggest that Gli3 repressor prevents abnormal neural tube patterning when *Gli1* is ectopically expressed from the *Gli2* locus, and the reduction in Gli3 repressor likely contributes to the dorsal expansion of the ventral neural progenitor domains of the *Gli2^1ki^*^/+^*;Ift88*^−/−^ double mutant neural tube.

### 3.5. Gli1 Expression from the Gli2 Locus Failed to Alter the Smo Mutant Phenotype

The ectopic moderate activation of the Hh pathway in the *Gli2^1ki^*^/+^*;Ift88*^−/−^ neural tube was in striking contrast to the *Ift88*^−/−^ mutant neural tube where both high and intermediate levels of Hh pathway activities were compromised [16], suggesting a functional difference between Gli1 and Gli2. It is possible that Hh signaling may activate Gli1 independent of the cilia. Alternatively, Gli1 may exhibit Hh-independent basal activity that is sufficient to drive ventral neural tube cell fates in more dorsal regions with the reduction in Gli3 repressor. We hypothesized that if Gli1 exhibited a Hh-independent activity, expressing *Gli1* from the *Gli2* locus should allow some ventral neural cell fates in the absence of *Smo*. To test this hypothesis, we analyzed the patterning of the *Gli2^1ki^;Smo* double mutant neural tube.

**Figure 4:**
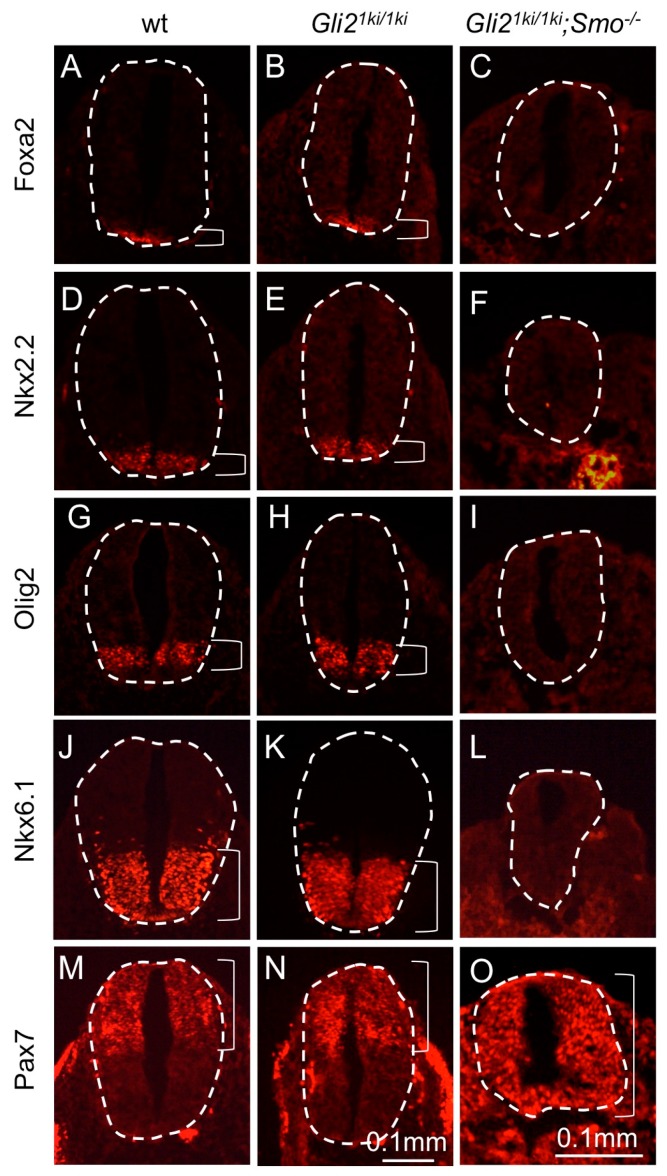
Expression of *Gli1* from the *Gli2* locus did not change neural tube patterning in the absence of Smo. Transverse sections of E10.5 embryos processed for immunofluorescence analyses. (**A**–**C**) *Foxa2* was expressed in the floor plates of the wild type (**A**) and *Gli2^1ki/1ki^* (**B**), but not in the *Gli2^1ki/1ki^*;*Smo*^−/−^ (**C**) neural tubes. (**D**–**F**) *Nkx2.2* labeled V3 interneurons in the wild type (**D**) and *Gli2^1ki/1ki^* (**E**), but was absent in *Gli2^1ki/1ki^*;*Smo*^−/−^ (**F**) neural tubes. (**G**–**I**) *Olig2* labels motor neuron progenitors in the wild type (**G**) and *Gli2^1ki/1ki^* (**H**), but not in *Gli2^1ki/1ki^*;*Smo*^−/−^ (**I**) neural tubes. (**J**–**L**) *Nkx6.1* was expressed in the wild type (**J**) and *Gli2^1ki/1ki^*(**K**), but not in *Gli2^1ki/1ki^*;*Smo*^−/−^ (**L**) neural tubes. (**M**–**O**) *Pax7* was expressed in the dorsal region of the wild type (**M**) and *Gli2^1ki/1ki^*(**N**), and was expanded ventrally in the *Gli2^1ki/1ki^*;*Smo*^−/−^ (**O**) neural tubes. Dashed lines outline the neural tubes and brackets mark the expression domains. Note the *Gli2^1ki/1ki^*;*Smo*^−/−^ images are of higher magnification to show the details of the neural tubes that are significantly smaller than those of their littermates. *n* = 3 embryos for each genotype.

*Smo*^−/−^ mutants were significantly smaller than control littermates at E9.5 and died shortly after [40]. *Gli2^1ki/1ki^;Smo*^−/−^ double mutant embryos were morphologically identical to those of *Smo*^−/−^ mutants (data not shown). In the neural tube, replacing both copies of *Gli2* with *Gli1* did not alter the expression of the floor plate marker *Foxa2* (Figure 4A,B), V3 interneuron marker *Nkx2.2* (Figure 4D,E), motor neuron progenitor marker *Olig2* (Figure 4G,H) and *Nkx6.1*, which marked V1, V2, V3 interneurons, motor neurons and floor plate (Figure 4J,K). Consistent with the morphological similarity, *Gli2^1ki/1ki^;Smo*^−/−^ exhibited loss of the floor plate (Figure 4C), V3 interneurons (Figure 4F), motor neurons (Figure 4I), V1 and V2 neurons (Figure 4L), similar to *Smo*^−/−^ mutants [40]. On the other hand, the dorsal neural tube marker *Pax7* (Figure 4M,N) was expanded ventrally in *Gli2^1ki/1ki^;Smo*^−/−^ neural tubes (Figure 4O). These observations indicated that Gli1 was not activated independent of Hh signaling, suggesting that the cilia-independent partial activation of Gli1 was dependent on Hh signaling.

### 3.6. Increased Protein Level Was Not the Major Reason for the Cilia-Independent Gli1 Activation

**Figure 5:**
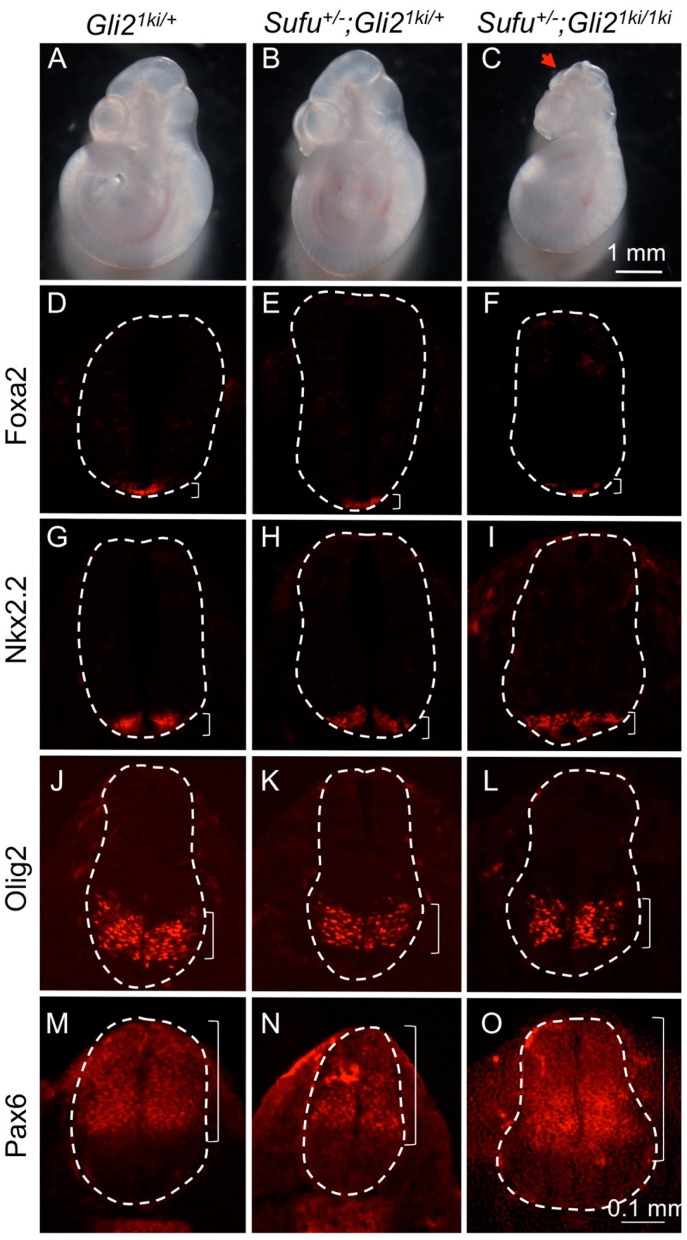
Reducing the dosage of Sufu did not alter the patterning in the *Gli2^1ki^* neural tube. (**A**–**C**) Lateral views of E10.5 embryos. *Gli2^1ki^*^/+^ (**A**) *Gli2^1ki^*^/+^*;Sufu*^+/−^ (**B**) embryos looked similar. One out of 4 *Gli2^1ki/1ki^;Sufu*^+/−^ embryos (**C**) exhibited midbrain exencephaly (arrowheads). (**D**–**O**) Transverse sections of E10.5 embryos processed for immunofluorescence analyses. (**D**–**F**) *Foxa2* was expressed in the floor plates of the *Gli2^1ki^*^/+^ (**D**), *Gli2^1ki^*^/+^*; Sufu*^+/−^ (**E**) and *Gli2^1ki/1ki^;Sufu*^+/−^ (F) neural tubes. (**G**–**I**) *Nkx2.2* labeled V3 interneurons in the *Gli2^1ki^*^/+^ (**G**), *Gli2^1ki^*^/+^*; Sufu*^+/−^ (**H**) and *Gli2^1ki/1ki^;Sufu*^+/−^ (**I**) neural tubes. (**J**–**L**) *Olig2* labeled motor neuron progenitors in the *Gli2^1ki^*^/+^ (**J**), *Gli2^1ki^*^/+^*; Sufu*^+/−^ (**K**) and *Gli2^1ki/1ki^;Sufu*^+/−^ (**L**) neural tubes. (**M**–**O**) *Pax6* was expressed in the dorsal and lateral regions of the *Gli2^1ki^*^/+^ (**M**), *Gli2^1ki^*^/+^*; Sufu*^+/−^ (**N**) and *Gli2^1ki/1ki^;Sufu*^+/−^ (**O**) neural tubes. Dashed lines outline the neural tubes and brackets mark the expression domains. *n* = 3 embryos for each genotype unless otherwise mentioned in the text.

Gli1 is resistant to both Cul1-mediated proteolytic processing and Cul3-mediated degradation, making it a more stable protein than Gli2 and Gli3 [41,42,43,44]. It is possible that replacing Gli2 with the more stable Gli1 protein in *Gli2^1ki^* mutant embryos brings a challenge to the Sufu-based negative regulation of Hh signaling, lowering the threshold for Hh pathway activation. If this is true, reducing the dosage of Sufu in the presence of ectopic *Gli1* expression, as in the *Gli2^1ki^*^/+^*;Sufu*^+/−^ and *Gli2^1ki/1ki^;Sufu*^+/−^ neural tubes, should lead to excess Hh pathway activation and dorsal expansion of some ventral progenitor domains. Our results showed that *Gli2^1ki^*^/+^*;Sufu*^+/−^ embryos were morphologically normal at E10.5 (Figure 5A,B, *n* = 6), whereas one out of 4 *Gli2^1ki/1ki^;Sufu*^+/−^ embryos exhibit midbrain exencephaly (Figure 5C). In the neural tube, the floor plate (Figure 5D–F), V3 interneuron (Figure 5G–I) and motor neurons (Figure 5J–L) were all formed in their proper D/V locations in the *Gli2^1ki^*^/+^*;Sufu*^+/−^ and *Gli2^1ki/1ki^;Sufu*^+/−^ double mutant neural tubes. Furthermore, *Pax6* expression domains in the *Gli2^1ki^*^/+^, *Gli2^1ki^*^/+^*;Sufu*^+/−^ and *Gli2^1ki/1ki^;Sufu*^+/−^ neural tubes were similar in size and location (Figure 5M–O), suggesting that the overall level of Gli proteins in *Gli2^1ki^*^/+^ mutant neural tube was not high enough to override the inhibitory function of Sufu and change the D/V patterning.

**Figure 6:**
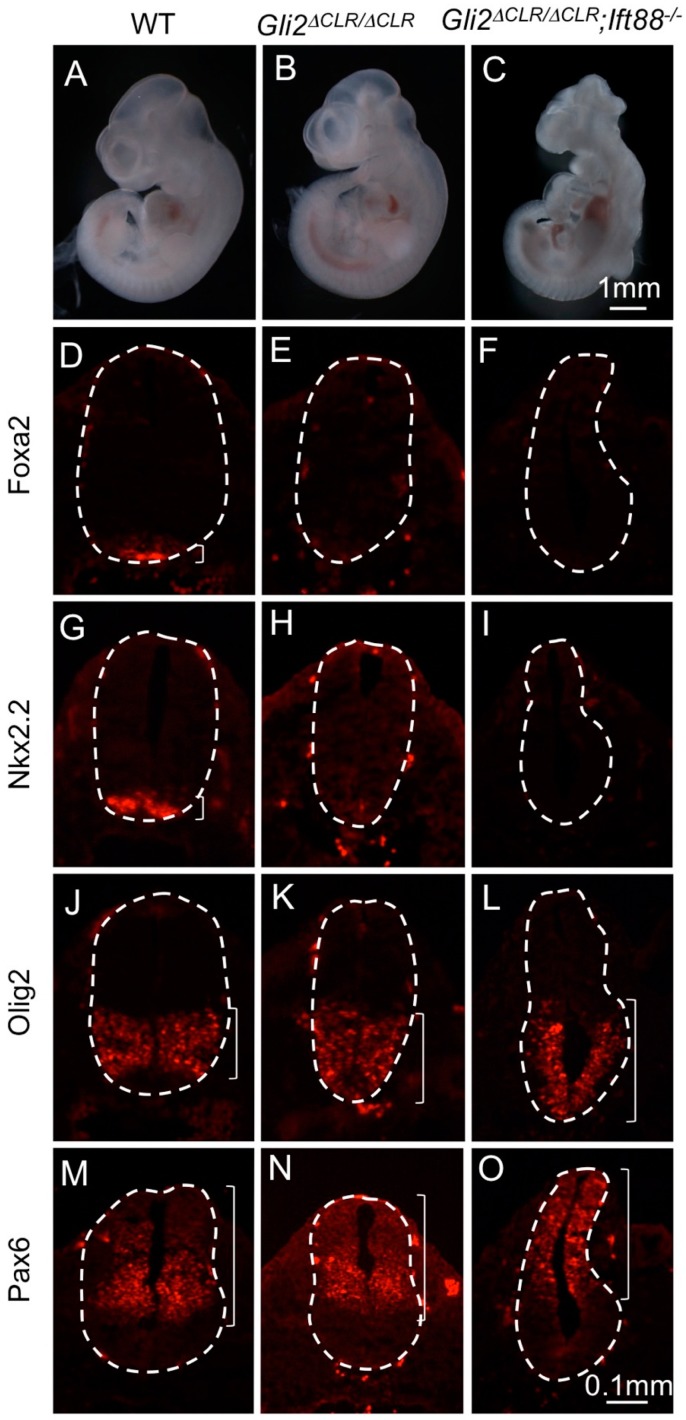
Gli2^∆CLR^ failed to activate ectopic Hh signaling in the absence of cilia. (**A**–**C**) Lateral views of E10.5 embryos. Wild type (**A**) and *Gli2^∆CLRki/∆CLRki^* (**B**) embryos looked similar. *Gli2^∆CLRki/∆CLRki^;Ift88*^−/−^ embryo (**C**) exhibited exencephaly and twisted body. (**D**–**O**)Immunofluorescent images of transverse sections through the E10.5 neural tubes. (**D**–**F**) *Foxa2* labeled floor plates in the wild type (**D**) neural tube. It was absent in the *Gli2^∆CLRki/∆CLRki^* (**E**) and *Gli2^∆CLRki/∆CLRki^;Ift88*^−/−^ (**F**) mutant neural tubes. (**G**–**I**) *Nkx2.2* was expressed in the V3 interneurons of the wild type neural tube (**G**) but was absent in the *Gli2^∆CLRki/∆CLRki^* (**H**) and *Gli2^∆CLRki/∆CLRki^;Ift88*^−/−^ (**I**) mutant neural tubes. (**J**–**L**) *Olig2* was expressed in motor neuron progenitors in the wild type neural tube (**J**). It was expanded ventrally in the *Gli2^∆CLRki/∆CLRki^* (**K**) and *Gli2^∆CLRki/∆CLRki^;Ift88*^−/−^ (**L**) mutant neural tubes. (**M**–**O**) *Pax6* was excluded from the ventral-most regions of the wild type (**M**), *Gli2^∆CLRki/∆CLRki^* (**N**) and *Gli2^∆CLRki/∆CLRki^;Ift88*^−/−^ (**O**) mutant neural tubes. Dashed lines outline the neural tubes. Brackets show the expression domains. *n* = 3 for each genotype.

We recently generated a mouse strain in which *Gli2* was replaced by *Gli2^∆CLR^*, a variant that did not enter the cilia but retained its transcriptional activity in the absence of *Sufu* [20]. Interestingly, the level of Gli2^∆CLR^ was higher than full-length Gli2 in E10.5 embryos, enabling us to directly test whether moderately increasing the level of Gli proteins was sufficient for a moderate activation of Hh signaling in the lateral regions of the neural tube in the absence of cilia. As shown in Figure 6, E10.5 *Gli2^∆CLRki/∆CLRki^* mutants were similar to wild type littermates morphologically (Figure 6A,B), whereas *Gli2^∆CLRki/∆CLRki^;Ift88*^−/−^ double mutant embryos exhibited exencephaly, twisted body and other morphological defects reminiscent of *Ift88*^−/−^ mutants (Figure 6C, compare to Figure 2C). In the neural tube, *Gli2^∆CLRki/∆CLRki^* mutants exhibited a reduction of the floor plate (Figure 6D,E) and V3 interneurons (Figure 6G,H). On the other hand, *Olig2*-expressing motor neuron progenitors expanded ventrally in these embryos (Figure 6J,K), whereas *Pax6* expression remained unchanged (Figure 6M,N). Different from *Gli2^1ki^*^/+^*;Ift88*^−/−^ mutants in which *Nkx2.2* and *Olig2* expression domains were expanded dorsally, the expression of *Foxa2* and *Nkx2.2* was absent in *Gli2^∆CLRki/∆CLRki^;Ift88*^−/−^ double mutant neural tube (Figure 6F,I), and the *Olig2* expression was expanded ventrally (Figure 6L). The only sign of moderate rescue of the ventral neural fate in the *Gli2^∆CLRki/∆CLRki^;Ift88*^−/−^ neural tube was the absence of *Pax6* expression in the ventral-most part of the neural tube, in contrast to the expression of *Pax6* throughout the *Ift88*^−/−^ mutant neural tube (Figure 6O, compared to Figure 2G) [16,18]. These results indicated that Gli2^∆CLR^, albeit exhibiting higher stability than wild type Gli2, did not activate Hh pathway ectopically as did Gli1 in the absence of cilia.

**Figure 7:**
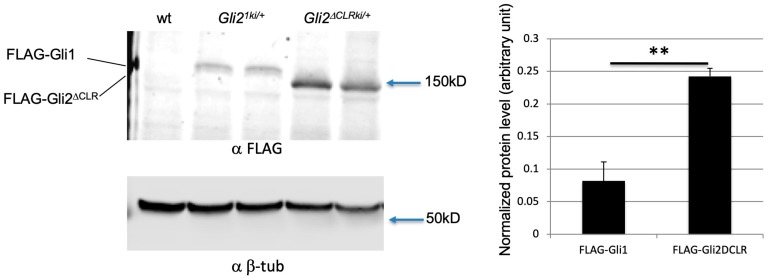
The level of Gli1 in *Gli2^1ki^*^/+^ embryos was lower than that of Gli2*^∆^*^CLR^ in *Gli2^∆CLRki^*^/+^ embryos. Immunoblots of E10.5 whole embryo lysates with antibodies against FLAG and β-tubulin. **: *p* = 0.0018 in two-tailed student *t*-test.

One potential explanation for the difference between the *Gli2^1ki^*^/+^*;Ift88*^−/−^ and *Gli2^∆CLRki/∆CLRki^;Ift88*^−/−^ neural tubes was that the level of Gli1 in the former was higher than that of Gli2^∆CLR^ in the latter. As a FLAG tag was introduced to the N-termini of both proteins in the knock-in embryos [32], we directly compared the expression levels of these two proteins in E10.5 embryos through immunoblot analyses. We found that the level of Gli2^∆CLR^ in *Gli2^∆CLRki^*^/+^ embryos was nearly three fold of that of Gli1 in *Gli2^1ki^*^/+^ embryos (Figure 7, *n* = 4 embryos for each strain). This result suggested that the surprising activation of the Hh signaling in the lateral neural tube of *Gli2^1ki^*^/+^*;Ift88*^−/−^ was not simply the result of higher Gli1 protein level.

## 4. Discussion

**Figure 8:**
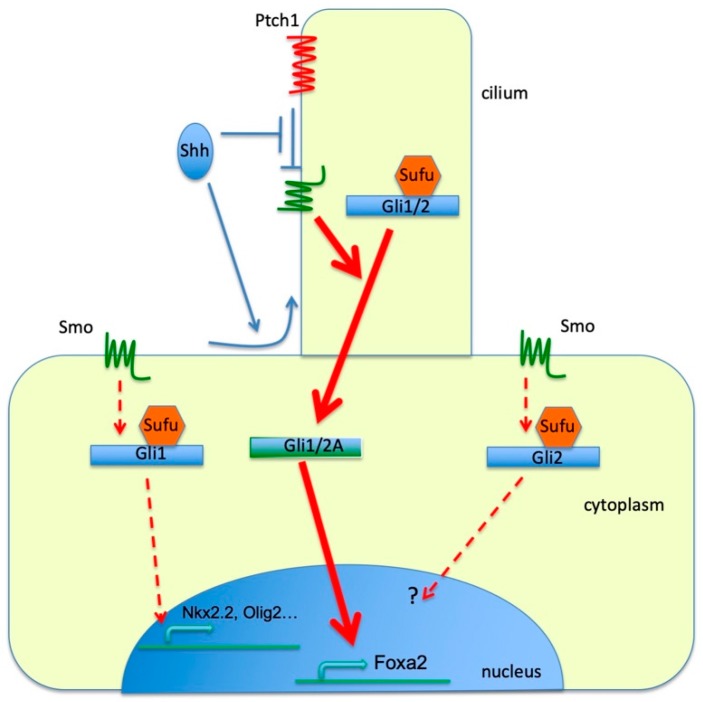
Cilia-dependent and independent activation of Gli proteins. Both Gli1 and Gli2 are activated by Smo-mediated Sonic hedgehog (Shh) pathway inside the cilia, leading to maximal activation (thick arrows) of target gene transcription (Foxa2). Outside of the cilia, Smo partially activates Gli1 and lower levels of transcriptional response (arrows with dashed lines, Nkx2.2 and Olig2). There is no direct evidence for cilia-independent Gli2 activation, but this possibility cannot be completely ruled out yet.

In this study, we compare the relationships between the cilia and two Gli family proteins, Gli1 and Gli2, in mouse neural tube patterning. Previous studies have shown that these relationships could not be addressed properly by in vitro overexpression due to the override of Sufu-mediated negative regulation [28,29]. To address this question properly, we compared neural tube patterning between *Ift88* mutant and *Gli2^1ki^;Ift88* double mutants. We found that although the cilia were required for the full activation of both Gli1 and Gli2, and the formation of the floor plate, they were not required for V3 interneuron and motor neuron progenitor formation when *Gli1* was expressed from the *Gli2* locus, suggesting that Gli1 activation was not fully dependent on the cilia (Figure 8).

Genetic analyses using *Ift88* or *Kif3a* mutants have been widely used to test the roles of cilia in numerous biological processes (e.g., [45,46,47]). Although Ift88 has been shown to regulate mitotic spindle orientation, immune synapse, and cell migration independent of the cilia, to our best knowledge, no solid evidence exists to support a cilia-independent role for Ift88 in Shh-mediated neural tube patterning [48,49,50,51]. Therefore, we strongly believe that the neural tube patterning changes in the absence of *Ift88* likely indicate the roles of the primary cilia in the activation of various Gli variants, rather than a cilia-independent function of *Ift88*.

One possible explanation for this cilia-independent partial Gli1 activation was that Gli1 was more stable than Gli2, leading to either Hh-independent activation of target gene expression or a lower threshold of cellular response to Hh signaling. Our results appeared to counter this explanation. First, we did not see any difference between *Smo* and *Gli2^1ki^;Smo* double mutant neural tube, suggesting that Gli1 was not activated in the absence of upstream Hh pathway input (Figure 8). Second, reducing the dosage of Sufu in *Gli2^1ki^* embryos did not result in an increase in Hh pathway activity and a change in neural tube patterning, suggesting that it was unlikely that increased Gli1 level led to a lower threshold to Hh response through overriding Sufu function. Finally, we show that Gli2^∆CLR^, with a protein level significantly higher than that of Gli1 in their respective knock-in embryos, did not support ectopic V3 and motor neuron progenitor formation in the absence of cilia, suggesting that the phenotype in *Gli2^1ki^*^/+^*;Ift88*^−/−^ double mutants did not result from a simple increase in Gli protein level.

Interestingly, *Gli2^1ki^* neural tube exhibits normal pattern along its D/V axis whereas V3 and motor neuron domains were expanded dorsally in *Gli2^1ki^;Ift88* double mutants, suggesting a negative role of the cilia in Hh signaling. We show that reducing Gli3 dosage in *Gli2^1ki^* neural tube similarly resulted in dorsal expansion of these ventral neuronal domains, suggesting that reduced Gli3 repressor activity in the absence of cilia contributed to this negative role of the cilia. Similar negative roles of the cilia have been reported in previous studies of skin and brain tumors caused by activating mutations in Gli proteins [45,46].

One remaining question is why Gli1, but not Gli2, appears to be partially activated in the absence of cilia if the elevated protein level is not likely the major contributing factor. It is possible that Gli1 has a unique response to Hh signaling outside of the cilia. Alternatively, the difference may be quantitative as Gli1 does not appear to have a repressor domain at its N-terminus, making it a much stronger transcriptional activator [3]. Therefore, if a cilia-independent activation mechanism exists to activate Gli proteins at a very low level, the effect should be more detectable with Gli1 (Figure 8). Although such a cilia-independent pathway for Gli-mediated transcription has not been detected, a non-canonical, transcription-independent Hh/Smo pathway appears to be independent of the cilia [52,53,54]. Further investigation will be needed to reveal the detailed molecular mechanism of the cilia-independent Gli1 activity.
